# Supplementary figures and images for: Anxiety and risk-taking behavior maps onto opioid and alcohol polysubstance consumption patterns in male and female mice
Source: bioRxiv. 2025 Sep 25:2024.08.22.609245. Originally published 2024 Aug 23. Preprint. [Version 2] doi: 10.1101/2024.08.22.609245 (PMC11370560; doi:10.1101/2024.08.22.609245)

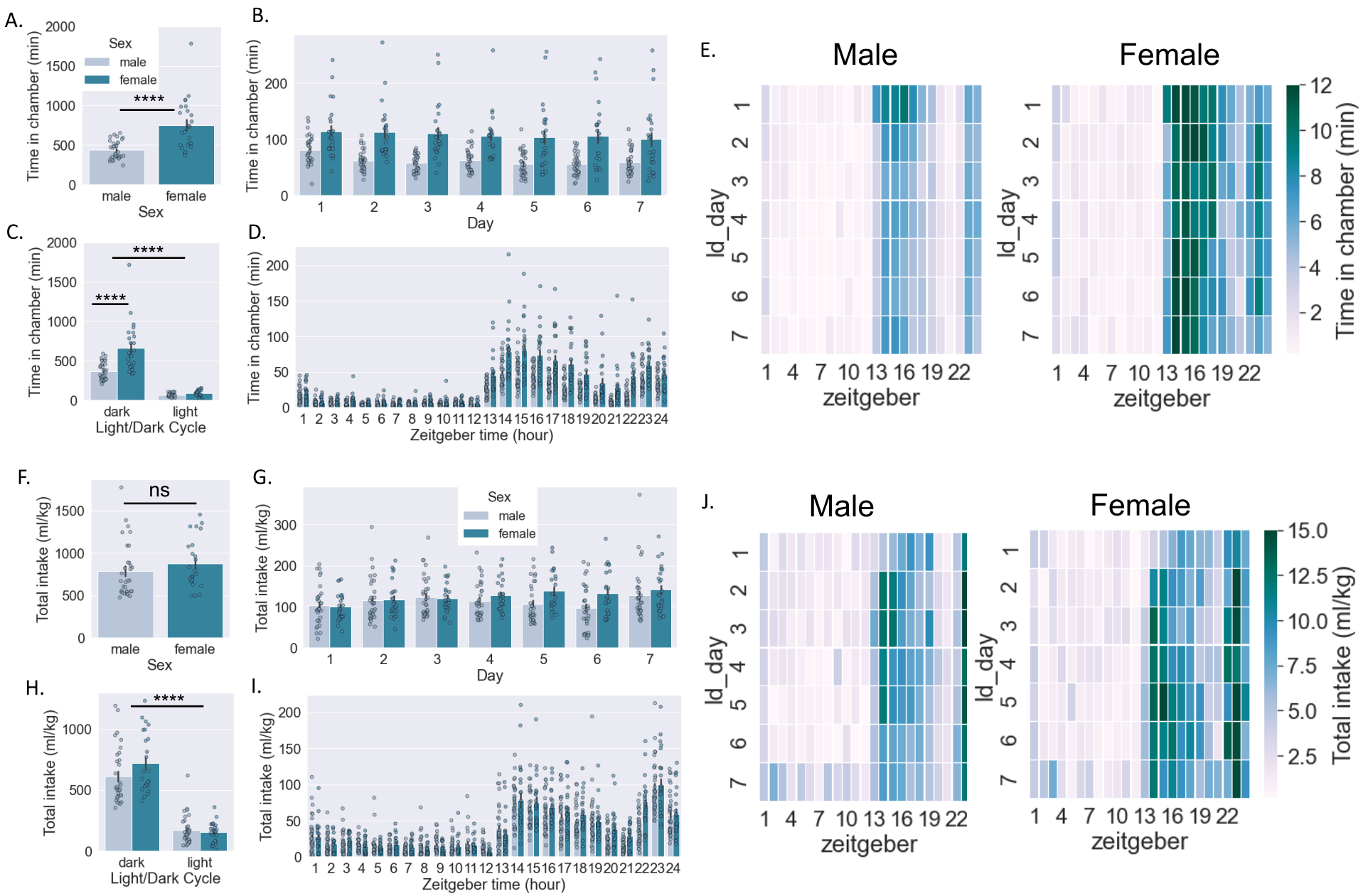

Supplement: Supplement 2 — Supp Figure 1: Activity and consumption in combined drinking chambers by sex A-E: Time spent in the drinking chambers in male and female mice in total (a), across days (b), across light/dark cycle (c), and across Zeitgeber time (d); heatmaps shown in e. F-J: Amount of liquid consumed by male and female mice (normalized to body weight) in total (f), across days (g), across light/dark cycle (h), and across Zeitgeber time (i); heatmaps shown in j. Student’s t-test (a,f); Two-way RM ANOVA post hoc BMCT (b-d. g-h). **p ≤ 0.01, ****p ≤ 0.0001. Values represent mean ± SEM. [file media-2.pdf]

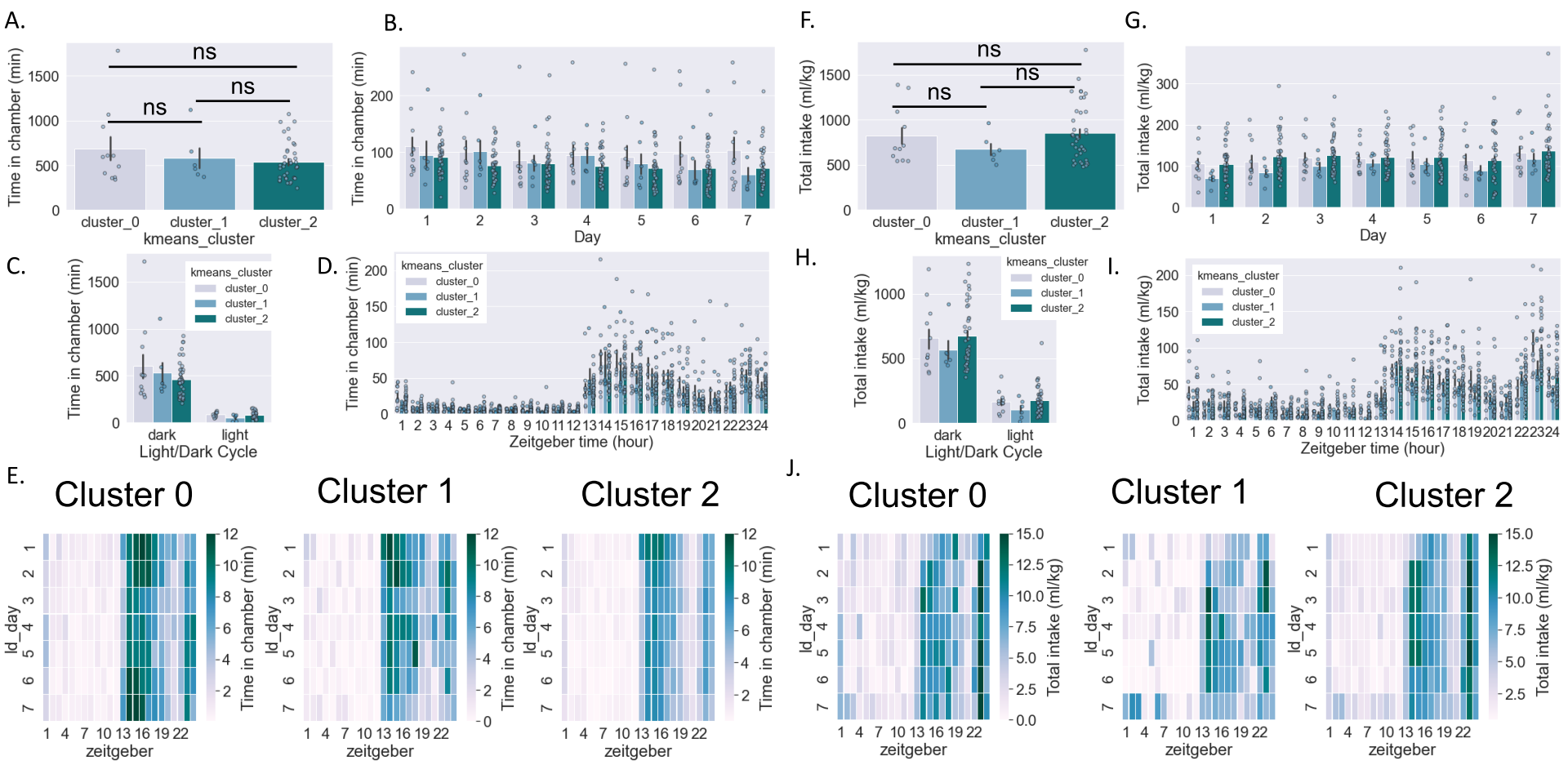

Supplement: Supplement 3 — Supp Figure 2: Activity and consumption in combined drinking chambers by cluster A-E: Time spent in the drinking chambers for each cluster in total (a), across days (b), across light/dark cycle (c), and across Zeitgeber time (d); heatmaps shown in e. F-J: Amount of liquid consumed by mice in each cluster (normalized to body weight) in total (f), across days (g), across light/dark cycle (h), and across Zeitgeber time (i); heatmaps shown in j. Student’s t-test (a,f); Two-way RM ANOVA post hoc BMCT (b-d. g-h). **p ≤ 0.01, ****p ≤ 0.0001. Values represent mean ± SEM. [file media-3.pdf]
